# Sleep quality and emotional reactivity in patients with borderline personality disorder

**DOI:** 10.3389/frsle.2024.1394979

**Published:** 2024-09-20

**Authors:** Valentina Socci, Fabiana Festucci, Tommaso Barlattani, Federico Salfi, Giulia D'Aurizio, Rodolfo Rossi, Michele Ferrara, Alessandro Rossi, Francesca Pacitti, Daniela Tempesta

**Affiliations:** ^1^Department of Biotechnological and Applied Clinical Sciences, University of L'Aquila, L'Aquila, Italy; ^2^Department of Systems Medicine, University of Rome Tor Vergata, Rome, Italy

**Keywords:** borderline personality disorder, sleep disturbances, sleep quality, emotion regulation, emotional reactivity

## Abstract

**Background/objective:**

Emotional dysregulation is bidirectionally associated with sleep disturbances, with potentially critical implications for emotional reactivity, in patients with borderline personality disorder (BPD). This study evaluated subjective and objective sleep quality, emotional regulation, and emotional reactivity in 20 patients with BPD compared to 20 non-clinical individuals.

**Methods:**

Subjective and objective sleep quality was assessed using the Pittsburgh Sleep Quality Index and a 3-day actigraphic measurement. Emotional regulation was evaluated using the Difficulties in Emotion Regulation Scale and the Berkeley Expressivity Questionnaire. Furthermore, each participant underwent an emotional reactivity task selected from the International Affective Picture System.

**Results:**

Compared to control subjects, individuals with BPD reported poor subjective sleep quality and objective sleep continuity disturbances, with more sleep fragmentation and decreased sleep efficiency. Moreover, BPD patients showed emotional dysregulation and altered subjective reactivity to emotional stimuli, particularly positively valenced stimuli.

**Conclusion:**

These results suggest the importance of further clarifying the specific direction of sleep-dependent emotional modulation in individuals with BPD, with significant clinical implications for patients with co-occurring sleep disturbances.

## 1 Introduction

In the last few decades, increasing evidence has suggested a complex and bidirectional interplay between sleep and emotions. Emotional states may affect subsequent sleep quality in different ways, including difficulties falling asleep and overnight sleep disruption, while sleep quality significantly affects consecutive emotional reactivity (Altena et al., 2016; Kahn et al., 2013). Sleep loss is commonly associated with mood changes, increased subjective irritability, and affective volatility (Rosen et al., 2006). Altered emotional processing has been consistently reported among the detrimental consequences of sleep loss and poor sleep quality for the emotional brain (Cunningham et al., 2022). At the neural level, sleep loss is associated with an amplified limbic response to negative stimuli, indicating a failure of top-down prefrontal control over emotional areas (Yoo et al., 2007).

Consequently, a critical effect of sleep loss on emotional processing could be the next-day enhancement of negative affective tone, with important implications for real-world settings (Zohar et al., 2005; Tempesta et al., 2018). In this respect, behavioral investigations have indicated a propensity of sleep-deprived individuals to assess experimental stimuli as more negative (Tempesta et al., 2010). Moreover, such emotional bias also emerged in poor sleepers, who showed more negative evaluations of neutral stimuli compared to good sleepers (Tempesta et al., 2015). Therefore, sleep loss could result in a negative bias in the categorization of emotionally unloaded stimuli (i.e., neutral pictures), an increased emotional (subjective and autonomic) reaction to these stimuli, or even heightened attention to negative stimuli (Tempesta et al., 2010). Collectively, these results point to sleep as a critical modulator of the emotion regulation process (Tempesta et al., 2018).

Emotional dysregulation is considered a defining feature of borderline personality disorder (BPD), a condition characterized by instability in interpersonal relationships, self-image, behavior, and affective regulation, associated with dangerous behaviors, self-harm, and suicidality. The lifetime prevalence of the disorder is ~2.8%, while patients with BPD account for 15%−28% in hospitals or psychiatric outpatient clinics (American Psychiatric Association, 2013). Due to its pervasive nature, BPD is associated with several chronic conditions that may significantly affect the quality of life, including sleep disturbances (Jenkins et al., 2022).

According to one of the most influential models of BPD development, emotional dysregulation represents the core symptom of the disorder, sustained by a constant interaction between a biological vulnerability and an invalidating environment (Linehan, 2005). Emotional vulnerability in BPD emerges early in life as a heightened emotional reactivity to environmental stimuli, particularly emotions in others. In the last few decades, research investigating emotional processing in BPD patients has partially supported the clinical observations that suggest an altered emotional reactivity in individuals with BPD, although with some inconsistencies. Compared to control subjects, a heightened startle response to unpleasant stimuli (Ebner-Priemer et al., 2005; Glenn and Klonsky, 2009) has been reported. Neuroimaging investigations in individuals with BPD have indicated altered emotional processing, such as increased activation of the amygdala during emotion-related tasks, including emotionally valenced pictures (Herpertz et al., 2001), facial emotions (Donegan et al., 2003), and social-emotional pictures (Koenigsberg et al., 2009). Koenigsberg et al. (2009) observed no differences in valence and arousal levels between a BPD group and a non-patient group but did find different patterns of regional brain activation in BPD patients.

Overall, although the hyperreactivity hypothesis has not been fully supported by recent metanalytic investigations at both the behavioral and physiological levels (Bortolla et al., 2020), BPD patients could be characterized by a tendency to negatively appraise emotional stimuli, independent of their valence, and more negative affectivity at rest compared to control groups. BPD individuals may have an attentional bias toward processing negatively valenced experimental stimuli of different natures (Carpenter and Trull, 2013).

Importantly, emotional dysregulation, a defining feature of BPD, with a critical role in its phenomenology, is bidirectionally associated with sleep disturbances (Palagini et al., 2017; Goldschmied, 2019). Studies indicate that individuals with BPD may exhibit significant sleep disturbances compared to both healthy (Semiz et al., 2008; Schredl et al., 2012) and clinical populations, such as depressed controls (De la Fuente et al., 2004). BPD has been associated with several sleep abnormalities, including disturbances in continuity, reduced rapid eye movement latency, and nightmares (Hafizi, 2013). Although a marked discrepancy between objective and subjective sleep quality measures indicative of an altered perception of sleep quality has also been reported in these subjects (Philipsen et al., 2005; Jenkins et al., 2022), Oltmanns and Oltmanns (2015) showed differences in sleep parameters between BPD patients and healthy controls, including less total sleep time (TST), decreased sleep efficiency (SE), longer sleep onset latency, and more sleep fragmentation. Therefore, BPD may be independently associated with sleep disturbances. In this context, the detrimental consequences of poor sleep for optimal emotional reactivity processing may be additionally relevant for BPD, a clinical condition characterized by instability in affective regulation and, therefore, a particular susceptibility to the negative emotional effect of sleep loss.

Overall, the current research concerning the relationship between sleep disturbances and emotional regulation in BPD is mostly methodologically heterogeneous, with investigations focusing on specific age ranges or including individuals with BPD features rather than a formal diagnosis; furthermore, inconsistencies also emerge when considering emotional reactivity measures and sleep quality assessments.

This study aimed to evaluate subjective and objective sleep quality, emotional regulation, and emotional reactivity in a sample of consecutively admitted patients with BPD using emotionally valenced pictorial stimuli selected from the International Affective Picture Set (IAPS: Lang et al., 2005) to elicit emotional reactivity. We hypothesize that the presence of emotion dysregulation, poor sleep quality, and altered subjective emotional reactivity toward negative stimuli in patients with BPD compared to non-clinical subjects.

## 2 Methods

### 2.1 Participants

In total, 40 subjects (12 males and 28 females; mean age = 33.6 ± 12) were selected to participate in the study from both clinical and non-clinical populations.

The clinical sample included 20 subjects (six males and 14 females; mean age = 35 ± 11.7), with a *Diagnostic and Statistical Manual of Mental Health Disorders, Fifth Edition* (*DSM*-5; American Psychiatric Association, 2013) diagnosis of BPD consecutively admitted to the psychiatric unit of “San Salvatore” Hospital over 12 months. Participants in the BPD group had three or more BPD features, as assessed by the Structured Clinical Interview for the *DSM*-5, Research Version (First et al., 2015).

Patients who were unable to provide informed consent or complete the tasks due to the presence of language barriers, intellectual disability, or other cognitive deficits were excluded from participating in the study. The presence of a major medical illness, other psychiatric comorbidities, or current conditions (e.g., substance or alcohol abuse, current major depressive episode) were further exclusion criteria. All BPD patients were receiving pharmacological treatment at the time of inclusion in the study, with stable medications and dose levels for at least 3 months before the study.

The control group consisted of 20 healthy volunteers (eight males and 12 females; mean age = 32.4 ± 11.7) recruited via social media and local advertisements and were matched with the BPD group for age and sex. Participants in the control group had never received any mental health treatment or been diagnosed with any mental or sleep disorders based on subjective reports.

This study was approved by the institutional review board of the University of L'Aquila (protocol n. 34/2018) and performed according to the principle established by the Helsinki Declaration. The patients/participants provided written informed consent to participate in this study.

### 2.2 Measures

#### 2.2.1 Sleep assessment

##### 2.2.1.1 Pittsburgh Sleep Quality Index

The Pittsburgh Sleep Quality Index (PSQI) is a nine-item measure of global sleep quality over the past month (Curcio et al., 2013) that measures seven domains: Sleep Quality, Sleep Latency, Sleep Duration, Habitual Sleep Efficiency, Sleep Disturbances, Use of Sleeping Medications, and Daytime Dysfunction.

Items are rated on a scale from 0 (*never*) to 3 (*three or more times per week*) and can be added to create the total score, where a score of >5 indicates poor sleep quality (Buysse et al., 1989; Curcio et al., 2013).

##### 2.2.1.2 Actigraphy

Traditional actigraphic count recordings were obtained using the Actiwatch. Micro electro mechanical system (MEMS) accelerometry signals were obtained using the Geneactiv recorder (ActivInsights Ltd., Kimbolton, UK). Calculation of the sleep parameters was performed off-line using a custom-written MATLAB program with a graphical user interface (version 2018a, The MathWorks, Inc., Natick, Massachusetts, USA), using the default medium threshold setting. The program was obtained directly from the authors (Te Lindert and Van Someren, 2013) and represents a validated method for transforming accelerometry data into traditional actigraphic movement counts. From the actigraphic data, deriving three main variables was possible: TST, SE (%), and Wake After Sleep Onset (WASO). In addition, other variables were calculated: Time in Bed; Sleep Onset Latency, Assumed Sleep Time, Number of Wake Bouts, Mean Wake Bout Time, Number of Sleep Bouts, Mean Sleep Bout Time, Mobile Time, and Immobile Time (see Table 1).

**Table 1:** Means (and standard deviation, *SD*) of Pittsburgh Sleep Quality Index (PSQI) domains, actigraphic sleep parameters and emotional measures separately for the two groups (borderline personality disorder [BPD] group and control group).

	**BPD group**	**Control group**	***t***	***p***
	**Mean (** ***SD*** **)**	**Mean (** ***SD*** **)**		
**Pittsburgh Sleep Quality Index**			
Subjective sleep quality	1.58 (0.7)	1.1 (0.3)	−2.57	0.01
Sleep latency	2.10 (0.9)	1.3 (0.9)	−2.62	0.01
Sleep duration	0.94 (0.7)	0.95 (0.6)	0.01	ns
Habitual sleep efficiency	0.94 (1)	0.65 (0.5)	−1.03	ns
Sleep disturbances	1.68 (0.7)	1.25 (0.4)	−2.21	0.03
Use of sleeping medication	0.05 (0.2)	0 (0)	0.03	ns
Daytime dysfunction	1.57 (1.3)	0.5 (0.6)	−4.40	< 0.001
Global score	10.36 (3.5)	5.75 (2.3)	−4.78	< 0.001
**Actigraphic measures**			
Wake after sleep onset (min)	33.9 (15.5)	24.5 (10.5)	−2.21	0.03
Total sleep time (min)	405.5 (70.9)	415.4 (65.6)	0.45	ns
Sleep efficiency (%)	87.2 (6.1)	90.43 (2.9)	2.06	0.04
Time in bed (min)	507.27 (70.6)	496.49 (76.5)	−0.06	ns
Sleep onset latency (min)	12.79 (10.7)	10.61 (10.1)	−0.73	ns
Assumed sleep time (min)	448.8 (74.2)	439.3 (73.4)	0.008	ns
Number of wake bouts	34.3 (11.6)	34.5 (10.4)	0.15	ns
Mean wake bout time	0.96 (0.42)	0.72 (0.17)	−2.42	0.02
Number of sleep bouts	34.72 (11.5)	34.70 (10.5)	0.09	ns
Mean sleep bout time	15.3 (10.08)	14.9 (7.51)	−0.14	ns
Mobile time	141.9 (43)	147.1 (40.6)	−0.23	ns
Immobile time	301.6 (83.7)	297.3 (47.6)	0.15	ns
**Difficulties in emotion regulation scale**			
Nonacceptance of emotional responses	20.4 (5.6)	11.7 (4.3)	−5.3	< 0.001
Difficulty engaging in goal-directed behavior	17.6 (5.6)	12.2 (5.1)	−3.1	0.003
Impulse control difficulties	17.5 (7.4)	10.3 (4.1)	−3.7	< 0.001
Lack of emotional awareness	14.1 (4.6)	14.10 (3.1)	0.03	ns
Limited access to emotion regulation	24.9 (8.5)	13.5 (4.2)	−5.3	< 0.001
Lack of emotional clarity	14.2 (5.5)	9.5 (3.3)	−3.2	0.003
**Berkeley expressivity questionnaire**			
Negative emotionality	27.3 (7.2)	26.3 (5.9)	−0.4	ns
Positive emotionality	22.2 (3.5)	22.50 (3.4)	0.3	ns
Impulse strength	32.7 (6.9)	28.4 (5.8)	−2.12	0.04
Total score	10.8 (3.7)	5.6 (2.4)	−5.15	< 0.001

t-test analysis results (t- and p-values) are also shown.

ns, not significant.

#### 2.2.2 Emotional regulation assessment

##### 2.2.2.1 Difficulties in Emotion Regulation Scale

The Difficulties in Emotion Regulation Scale (DERS; Sighinolfi et al., 2010) consists of 36 multiple-choice items that measure characteristic individual patterns of emotion regulation.

The questionnaire contains six scales: (1) Non-Acceptance (non-acceptance of emotional responses), formed by the items that reflect the tendency to experience negative secondary emotions in response to one's negative emotions or have reactions of non-acceptance with respect to their discomfort; (2) Goals (difficulty in adopting goal-oriented behaviors) includes items that reflect the difficulties in concentrating and performing a task when experiencing negative emotions; (3) Impulse (control problems) reveals the difficulty in keeping control of their behavior when experiencing negative emotions; (4) Awareness (lack of emotional awareness) contains items that emphasize the tendency of paying attention to emotions and the relative ability to identify them (in the phase of calculating the score, the answers to this scale must be reversed); (5) Strategies (limited access to emotional regulation strategies) reflects the belief that effectively regulating emotions once they have occurred is particularly difficult; and (6) Clarity (a lack of emotional clarity) includes items that reflect the degree to which people can distinctly understand what emotion they are experiencing. High scores on the DERS are associated with both general emotional instability and difficulty in experiencing positive emotional states.

##### 2.2.2.2 Berkeley Expressivity Questionnaire

The Berkeley Expressivity Questionnaire (BEQ) is a 16-item self-report questionnaire useful for analyzing an individual's emotional regulation.

Gross and John (1995) focused on two parts of the generative process of emotions: the activation of emotional response tendencies and their subsequent modulation. The scales include both positive and negative emotions, such as sadness, fear, anger, and happiness. Through factor analysis, three subscales were identified: Impulse Strength, Positive Expressivity, and Negative Expressivity. Positive and Negative Expressivity refers to the degree to which both emotional response tendencies are expressed behaviorally. Impulse Strength, by comparison, represents the physical and behavioral changes that accompany emotional responses that are difficult to stop or hide.

Each item involves a response on a 7-point Likert scale, ranging from 1 (*strongly disagree*) to 7 (*strongly agree*). Three items are reverse-scored. Higher scores indicate greater expressiveness (Gross and John, 1995).

#### 2.2.3 Emotional reactivity task

Ninety color pictures from the IAPS (Lang et al., 2005) were selected, of which 30 were pleasant (mean valence: 8.0; mean arousal: 5.1; e.g., pictures of infants, happy family situations, natural landscapes, etc.), 30 neutral (mean valence: 5.0; mean arousal: 3.6; e.g., faces with neutral expressions, household objects, etc.), and 30 unpleasant (mean valence: 2.0; mean arousal: 6.0; e.g., spiders, mutilations, dead bodies, etc.).

The subjects were tested individually in a dimly lit room while seated in front of a 15-in. computer monitor at a distance of 50 cm. For each session, subjects were instructed that a series of 90 trials would be presented and that, for each trial, they would be asked to rate the affective valence and arousal of each picture.

The order of (pleasant–neutral–unpleasant) picture presentation was randomized for subjects. Trials started with a 2-s full-screen presentation of one picture. After a 1-s black mask, a display containing a smaller version of the same picture (located on the upper part) and the Self-Assessment Manikin (SAM) valence scale (located on the lower part) was presented (Bradley and Lang, 1994). The SAM valence scale consists of a cartoon-type figure showing nine human emotional expressions, ranging from smiling and happy to frowning and unhappy, are represented. This display remained visible until the participant responded or for 3 s. After the participant's valence rating, another display was presented with the SAM arousal scale, consisting of another cartoon-type figure showing nine expressions ranging from calm and relaxed to excited and wide-eyed. Similarly, the display remained visible until the participant responded or for 3 s. The valence and arousal ratings were made on a 9-point scale by pressing one of the keys of the PC keyboard, labeled from 1 to 9.

For each image, therefore, the subject indicated a score from 1 (*not very pleasant*) to 9 (*very pleasant*) to express the degree of pleasantness/unpleasantness of the image and, again for the same image, express a score from 1 (*not very activating*) to 9 (*very activating*) for the arousal sphere.

The presentation of stimuli and the recording of responses were managed using SuperLab 4.0 software for Windows (Cedrus Corporation, San Pedro, CA, USA).

### 2.3 Procedure

Informed consent was obtained from all participants before they took part in the study.

Participants were instructed to continuously wear the actigraph on their non-dominant wrist for 3 days. During the morning—between 10:00 a.m. and 12:00 p.m.—at the end of the 3 days of actigraphic recording, the previously described questionnaires (PSQI, DERS, and BEQ) and the emotional reactivity task were administered to each participant.

### 2.4 Statistical analyses

Of the 20 selected control subjects, a participant was not included in the statistical analysis due to the high miss-response rate in the tasks administered.

To assess our main hypothesis on the relationship between sleep quality and emotional reactivity, the mean valence and arousal ratings of the IAPS pictures were submitted to a mixed-model analysis of variance (ANOVA) with Group (BPD vs. control group), as between factors, and Affective Valence (positive vs. negative vs. neutral), as within factors. We report partial eta-squared ($\eta_{\text{p}}^{2}$) to document effect sizes.

When needed, *post hoc* Bonferroni tests were carried out.

Subjective (PSQI) and objective sleep measures (actigraphy), as well as emotional measures, were assessed using the independent samples *t*-test comparing the performance of the BPD and control groups. Cohen's *d* was computed to provide an effect size estimate for between-group comparisons.

Regarding the sleep measures, for the PSQI, we considered the mean of all seven subscales and the global score. For the actigraphy, we considered the mean of WASO (min), TST (min), and SE (%).

Moreover, concerning emotional scales, we considered the mean of all six DERS subscales, and all three BEQ subscales and the total score.

A *p*-level of < 0.05 was considered significant. All statistical analyses were performed using jamovi (version 2.3.21).

## 3 Results

### 3.1 Sleep measures

For a clearer and more schematic view of the scores, the reader is referred to Table 1.

#### 3.1.1 PSQI

The PSQI scores were significantly different between groups (*t*_37_ = −4.78, *p* < 0.001, *d* = 1.53), indicating higher PSQI scores (reflecting poorer sleep quality) of the BPD (10.36 ± 3.5) group compared to the control group (5.75 ± 2.3). In addition, the following clinical domains of sleep difficulties measured by the PSQI showed significant differences between the BPD and control groups: Subjective Sleep Quality (*t*_37_ = −2.57, *p* = 0.01, *d* = 0.82), Sleep Latency (*t*_37_ = −2.62, *p* = 0.01, *d* = 0.84), Sleep Disturbances (*t*_37_ = −2.21, *p* = 0.03, *d* = 0.70), and Daytime Dysfunction (*t*_37_ = −4.40, *p* < 0.001, *d* = 1.41). No other main effects were statistically significant.

#### 3.1.2 Actigraphy

The WASO scores were significantly different between the groups (*t*_37_ = −2.21, *p* = 0.03, *d* = 0.70). Indeed, the BPD group (33.9 ± 15.5) had a longer WASO compared to the control group (24.5 ± 10.5). In addition, the results showed a significant SE% (*t*_37_ = 2.06, *p* = 0.04; *d* = 0.66), indicating a lower sleep efficiency in the BPD group (87.2 ± 6.1) compared to the control group (90.43 ± 2.9). No significant effects were observed in TST.

Finally, the Mean Wake Bout Time scores were significantly different between groups (*t*_37_ = −2.42, *p* = 0.02, *d* = 0.78), showing a higher wake time in the BPD group compared to the control group (0.96 ± 0.42 and 0.72 ± 0.17, respectively).

### 3.2 Emotional measures

For a clearer and more schematic view of the scores, the reader is referred to Table 1.

#### 3.2.1 DERS

The following DERS subscales showed significant differences between BPD and control groups: Non-Acceptance (*t*_37_ = −5.3, *p* < 0.001, *d* = 1.72), Goals (*t*_37_ = −3.1, *p* = 0.003, *d* = 1), Impulse (*t*_37_ = −3.72, *p* < 0.001, *d* = 1.20), Strategies (*t*_37_ = −5.31, *p* < 0.001, *d* = 1.70), and Clarity (*t*_37_ = −3.2, *p* = 0.003, *d* = 1.02). No other main effects were statistically significant.

#### 3.2.2 BEQ

The BEQ Impulse Strength subscale score was significantly different between the BPD and control groups (*t*_37_ = −2.12, *p* = 0.04, *d* = 0.67), indicating that, compared to the control group, participants in the BPD group showed a lower ability to stop or hide behavior and physical activation following emotional stimuli elaboration. In addition, the analysis showed a significant effect on the BEQ total score (*t*_37_ = −5.15, *p* < 0.001, *d* = 1.65), which was highest for the BPD group compared to the control group. No other main effects were statistically significant.

##### 3.2.2.1 Emotional reactivity task: valence ratings

The ANOVA showed a main significant effect for Group (*F*_1, 37_ = 4.28, *p* = 0.04, $\eta_{\text{p}}^{2}$ = 0.11), indicating that valence ratings were significantly higher for the BPD group (mean ± *SE* = 4.58 ± 0.11) than the control group (mean ± *SE* = 4.26 ± 0.10). Moreover, the analysis revealed a significant main effect of Valence (*F*_1, 37_ = 877.1, *p* < 0.001, $\eta_{\text{p}}^{2}$ = 0.96), indicating that the neutral pictures (mean ± *SE* = 3.89 ± 0.2) were rated with lower arousal scores compared to the negative (mean ± *SE* = 6.16 ± 0.3) and positive (mean ± *SE* = 6.37 ± 0.2) pictures.

Finally, the Group × Valence interaction was also significant (*F*_1, 37_ = 3.8, *p* = 0.02, $\eta_{\text{p}}^{2}$ = 0.09). However, a *post hoc* comparison of the interaction means showed no differences between the two groups for positive, negative, and neutral valence.

##### 3.2.2.2 Emotional reactivity task: arousal ratings

The ANOVA showed a significant effect for Group (*F*_1, 37_ = 5.30, *p* = 0.02, $\eta_{\text{p}}^{2}$ = 0.13), indicating that arousal ratings were significantly higher for the BPD group (mean ± *SE* = 5.94 ± 0.2) than the control group (mean ± *SE* = 5 ± 0.2). Moreover, the analysis revealed a significant main effect of Affective Valence (*F*_1, 37_ = 36.8, *p* < 0.001, $\eta_{\text{p}}^{2}$ = 0.50), indicating that the neutral pictures (mean ± *SE* = 3.89 ± 0.2) were rated with lower arousal scores compared to the negative (mean ± *SE* = 6.16 ± 0.3) and the positive (mean ± *SE* = 6.37 ± 0.2) pictures.

**Figure 1:**
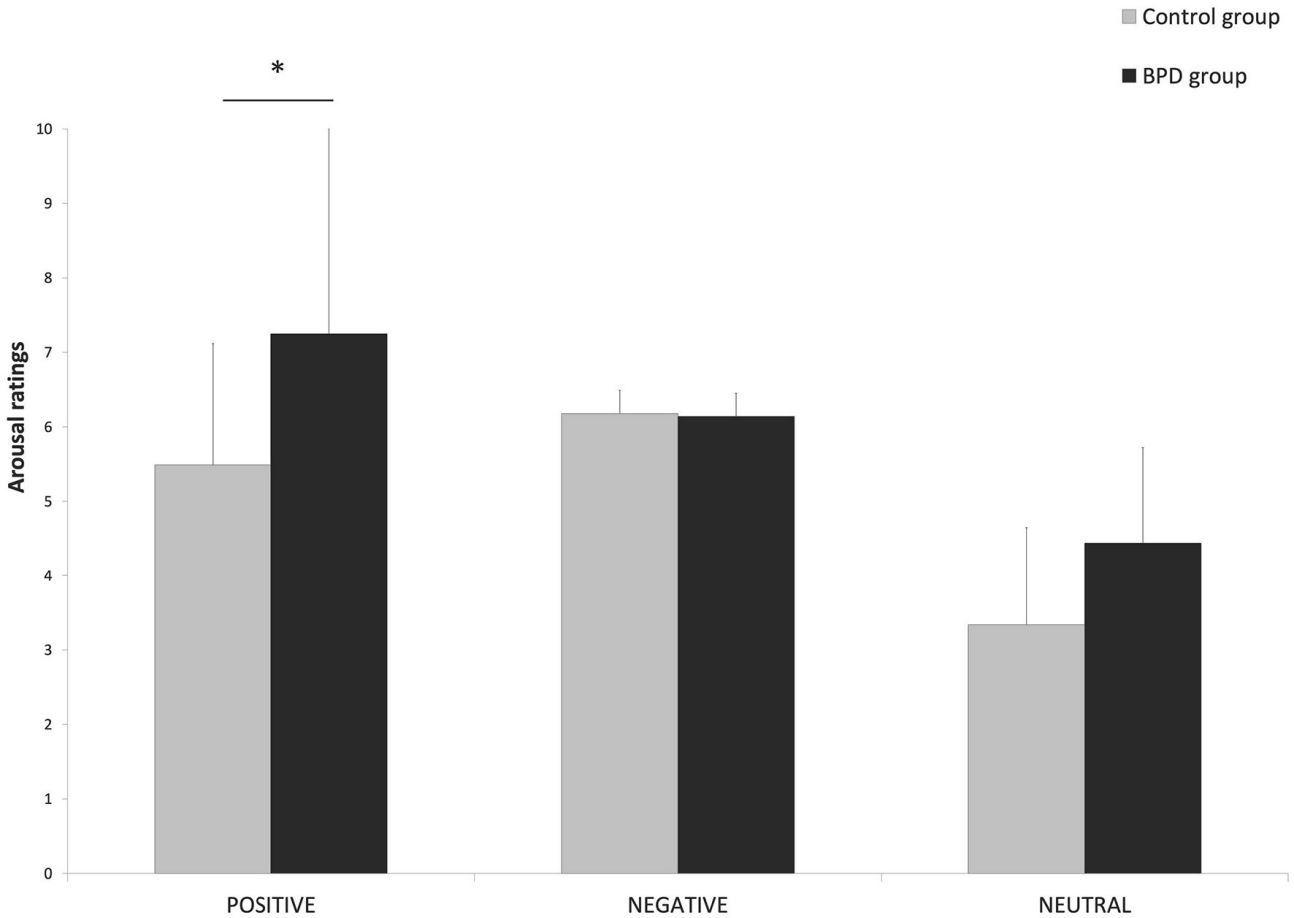
Mean scores (and *SD*) to the arousal ratings in the Emotional Reactivity Task for each of the two groups (BPD group and control group) as a function of valence (negative vs. positive vs. neutral). The asterisks indicate that the BPD group rated positive pictures as more activating than the control group (*p = 0.005).

Finally, the interaction effect Group × Valence was also significant (*F*_1, 37_ = 4.02, *p* = 0.02, $\eta_{\text{p}}^{2}$ = 0.1). *Post hoc* comparisons indicated that the BPD group (mean ± *SE* = 7.25 ± 0.3) rated positive pictures as more activating than the control group (mean ± *SE* = 5.49 ± 0.3, *p* = 0.005). Instead, for neutral pictures, there was only a tendency for the BPD group to evaluate arousal ratings higher (mean ± *SE* = 4.44 ± 0.2) compared to the control group (mean ± *SE* = 3.34 ± 0.2, *p* = 0.1). No difference was observed in the evaluation of the negative pictures between the two groups (*p* = 1) (Figure 1).

### 3.3 Correlations

Pearson's *r* coefficients were calculated to assess the relationships between sleep quality, emotional regulation, and emotional reactivity. For the whole group, the arousal rating for positive images was associated with the PSQI global score (*r* = 0.42, *p* < 0.001), the PSQI Sleep Disturbances domain (*r* = 0.36, *p* < 0.01), and the WASO actigraphic measure (*r* = 0.37, *p* < 0.01). Furthermore, arousal rating for positive stimuli was associated with the following emotional measure: the BEQ total score (*r* = 0.44, *p* < 0.01) and the Non-Acceptance (*r* = 0.38, *p* < 0.01), Goals (*r* = 0.46, *p* < 0.01), Impulse (*r* = 0.39, *p* < 0.01), and Strategy (*r* = 0.43, *p* < 0.01) DERS scales.

Moreover, significant associations emerged between the arousal rating for neutral images and the WASO (*r* = 0.40, *p* < 0.01), Goals (*r* = 0.38, *p* < 0.01), Impulse (*r* = 0.40, *p* < 0.01), and Strategy (*r* = 0.37, *p* < 0.01) DERS scales.

## 4 Discussion

In the present study, we evaluated subjective and objective sleep quality and emotional reactivity in a sample of individuals with BPD compared to a non-clinical sample. Overall, the results showed poorer sleep quality and alterations in emotional regulation and emotional reactivity in individuals with BPD.

Concerning subjective sleep quality, our results confirm previous evidence of self-reported poor sleep quality in BPD patients as evidenced by the PSQI scores. In particular, the BPD group reported higher global PSQI scores, indicative of poor sleep quality and an increased subjective sleep latency, sleep disturbances, and daytime dysfunction, such as sleepiness, compared to the non-clinical group. These results align with previous investigations showing a significantly impaired self-reported sleep quality in BPD individuals compared to control subjects (Semiz et al., 2008; Schredl et al., 2012), as well as other clinical samples (De la Fuente et al., 2004).

The differences observed in self-reported sleep quality in the clinical group were confirmed by the objective sleep quality assessment, specifically for the actigraphic SE% and WASO measures; in contrast, no differences emerged for the actigraphic TST measure. These results suggest that, compared to non-clinical populations, individuals with BPD may display sleep quality disturbances, such as reduced sleep efficiency and nighttime awakenings, even in the absence of marked differences in terms of objective sleep quantity. Overall, the actigraphic sleep quality measurement in this study confirms previous investigations that reported objective sleep continuity disturbances, including decreased sleep efficiency and more frequent arousals, in BPD individuals compared to controls (Oltmanns and Oltmanns, 2015).

Regarding the emotional measures included in the study, as expected, our data highlight an alteration in the emotion regulation process in BPD patients assessed using the DERS, as seen in the diminished capacity of response to negative emotional stimuli, in the alteration of the adopting a goal-oriented behavior and keeping control of actions when experiencing emotions negatively connoted. When experiencing negative emotions, the BPD subjects seem unable to cope with the situation: they reported feeling angry, ashamed, weak, and guilty about their negative emotional state. The DERS's goal-oriented subscale reported the BPD subjects' difficulty focusing on anything but the trigger that upset them. Regarding the BEQ assessment, while no differences emerged between groups for positive and negative behavioral emotional expressivity, our data highlight that the BPD group shows significantly worse control of impulse, both physically and behaviorally: these subjects stated experiencing intense emotions and body reactions; they seem unable to hide their feelings and stop crying and reported to feel out of control when upset.

These alterations in the emotional regulation process were partly confirmed by the emotional reactivity task results showing significantly higher valence and arousal ratings for emotional stimuli in individuals with BPD compared to the non-clinical sample. Furthermore, results showed higher arousal ratings for positively valenced emotional stimuli in BPD patients compared to controls, while no other difference emerged between the two groups when considering arousal ratings. Therefore, the current findings do not support the hypothesis of BPD hyper-responsiveness to unpleasant stimuli (Ebner-Priemer et al., 2005; Hazlett et al., 2007). Together, our results are indeed suggestive of a generalized alteration in the emotional reactivity process in individuals with BPD extending to all types of emotional stimuli; at the same time, results also indicate the presence of a more specific, valence-related alteration in the processing of emotional stimuli, with BPD rating positive images as more activating compared to controls. Similarly, a tendency to rate neutral images as more activating also emerged in the BPD group. Interestingly, correlational analysis performed in the whole sample showed that worse sleep quality was associated with higher arousal ratings for positive images and higher scores on emotional dysregulation measures, particularly on the BEQ scale and some DERS subscales (i.e., Non-Acceptance, Impulse, Goals, and Strategies). Therefore, these results suggest a link between sleep quality, emotional regulation abilities, and subjective emotional reactivity, particularly toward positive emotional stimuli. In this respect, while there is a substantial consensus concerning the crucial role of sleep for emotional processing and daytime emotional reactivity, the specific influence of sleep loss and sleep disturbances in affecting reactivity to different emotional events remains equivocal due to contrasting results (Tempesta et al., 2018). Further investigations, possibly including large samples and more ecological experimental stimuli, are therefore needed to clarify these complex relations.

The current research has several limitations. First, the main limitation concerns the relatively small sample size that circumscribes the generalizability of the results. In this respect, our analyses revealed low effect size measures for the observed effects in the emotional reactivity task probably linked to the limited sample size. Overall, these preliminary findings should be confirmed by future research to reach practical applications. Second, although well matched for age and gender, participants' mental health status in the non-clinical group was assessed using self-report measures so that the presence of clinically relevant actual mental health conditions potentially affecting the results cannot be entirely excluded. Similarly, emotional reactivity was assessed using subjective ratings with no concurrent objective measures. Also, the mean PSQI global score in the non-clinical sample indicates the possible presence of subclinical sleep disorders in this group, which may have impacted the results. Furthermore, pharmacological treatment in BPD patients potentially attenuating the subjective reactivity to negative stimuli should also be considered.

Nevertheless, these results indicate altered sleep quality and emotional reactivity in a naturalistic setting of BPD patients, partially overcoming the potential limitations of prolonged pharmacological and psychotherapeutic interventions. Therefore, these results are informative for sleep disorders and emotional dysregulation as potential targets in an acute setting. In addition, the results of this study are potentially relevant also for psychotherapeutic treatment, suggesting the importance of early detection and treatment of possible sleep disorders in individuals with BDP to promote optimal emotional reactivity, facilitate the acquisition of emotional regulation strategies, and maximize their effectiveness over time.

Future investigations should further elucidate the specific direction of sleep-dependent emotional modulation in patients with BPD, including more ecologically valid and sensitive experimental stimuli in conjunction with objective measures. Clarifying how sleep affects next-day emotional brain functioning is crucial for better understanding and treating affective disturbances in individuals with BPD.

## Data Availability

The raw data supporting the conclusions of this article will be made available by the authors, without undue reservation.
